# Recent Advances in AI and GenAI for Health Informatics

**DOI:** 10.3390/healthcare14040495

**Published:** 2026-02-14

**Authors:** Sio Iong Ao, Vasile Palade, Chris Holt, Suzy Araujo, Mike Gourlay, Danina Kapetanovic

**Affiliations:** 1 International Association of Engineers, Unit 1, 1/F, Hung To Road, Hong Kong; 2 Conrad School of Entrepreneurship & Business, University of Waterloo, Engineering Bldg. 7, 2316, Waterloo, ON N2L 3G5, Canada; christopher.holt@uwaterloo.ca; 3 Centre for Computational Science and Mathematical Modelling, Coventry University, Innovation Village 10, Coventry CV1 2TL, UK; ab5839@coventry.ac.uk; 4 Waterloo Regional Health Network (WRHN), 911 Queen’s Blvd, Kitchener, ON N2M 1B2, Canada; suzy.araujo@wrhn.ca (S.A.); mike.gourlay@wrhn.ca (M.G.); danina.kapetanovic@carenext.ca (D.K.); 5 University of Waterloo, Waterloo, ON N2L 3G5, Canada

**Keywords:** artificial intelligence, generative AI, large language models, health informatics, health analytics, clinical decision support, patient care, electronic health records, hospital management, remote patient monitoring

## Abstract

The emergence of large language models (LLMs) and generative artificial intelligence (GenAI) has marked a turning point in health informatics. AI has become a very helpful tool for health informatics applications, with numerous AI applications in health informatics being reported in the last years. The objective of this paper is to synthesize the common concerns and opportunities raised by recent popular reviews on AI and health informatics. The main methodological topics covered in this up-to-date review include traditional AI, GenAI, and LLMs. The literature search was conducted through the popular academic database Scopus, which covers over one hundred million records, including both computer science and healthcare. Among these popular reviews (measured by the number of citations that each one received), clinical decision support, patient care, electronic health records, hospital management, and remote patient monitoring are the most mentioned healthcare topics. Different from the majority of the existing reviews that narrowly cover on one to a few topics in healthcare, our review is designed with the objective to provide a broad coverage, such that practitioners may benefit from comprehensive insights covering the above mentioned five popular topics in AI health informatics applications. Based on an in-depth analysis of these reviews by human experts, the main AI tools used, their main challenges, and some future directions have been identified in our investigation. Patient privacy, cybersecurity, ethics, clinical accountability, engaging health professionals, benchmarks and standardization, as well as lack of explainability are the common concerns identified from the literature covered in this review.

## 1. Introduction

Health informatics denotes the deployment of digital tools to streamline healthcare processes, facilitate data interpretation, and support clinical judgments. Healthcare initiatives often focus on optimizing care delivery and enhancing patient outcomes through advanced digital technologies. Popular topics like decision support systems, predictive analytics, user-centered technologies, and ethical concerns are receiving growing attention in healthcare [1]. Another topic that is gaining popularity is the adoption of telehealth and mobile health technologies, especially following the onset of the COVID-19 pandemic [2].

AI demonstrates effectiveness across healthcare, including in early disease detection, drug discovery, treatment optimization, diagnostics, and decision support tools [3]. For example, in mobile health, AI is used for functions such as clinical decision support, patient education, health monitoring, remote consultations, and public health surveillance. Digital health tools like AI-driven wearable sleep monitors can help bridge knowledge gaps by disseminating reliable, evidence-based information to the public [4]. In medical imaging, AI holds the promise to boost performance and enhance patient care, as AI systems can process large volumes of imaging data and detect subtle patterns that may elude even experienced clinicians [5]. AI-driven tools are shown to be highly effective in interpreting diagnostic images like MRIs, CT scans, and X-rays. The AI application in radiological imaging has achieved diagnostic accuracy rates exceeding 90%, outperforming conventional approaches in identifying conditions like malignancies [6]. In diagnosing conditions, AI applications have also shown strong performance according to Lee and Kalpathy-Cramer [7].

AI tools become multifaceted digital aides for healthcare professionals [8]. AI is able to rapidly incorporate new medical information into its operational base, maintaining an up-to-date knowledge set that allows it to recommend the latest evidence-based treatments. In contrast, healthcare professionals must dedicate significant time to remain current with emerging medical literature, which may in turn limit their capacity to do so effectively [9]. AI can also enhance operational workflows by cutting down the time clinicians spend on repetitive managerial duties by as much as 70% in some tasks, according to Spatharou et al. [10].

This review investigates the contexts and details of the selected recent review articles on AI, GenAI, LLMs, and health informatics, such that practitioner audiences may benefit from more information about how these AI advances have already passed the pilot stages and have been implemented in many real-world health systems. Other names of traditional AI include narrow AI or weak AI, which refers to the intelligent execution of specific tasks [11]. GenAI may be regarded as the next AI generation that is capable of developing something new, while LLMs refer to AI, and in particular deep learning, for natural language processing for handling advanced human language-based tasks [12]. Section 2 describes our methods for identifying relevant recent review articles. In Section 3, we present the results of our literature searches and analysis. In Section 4, the focus is on the synthesis and discussion of the concerns in these reviews, such that high-level issues common across different health informatics topics are identified. Section 5 presents some conclusions. For the convenience of those readers who may like to have more information about the common terms in health informatics and AI, Appendix A presents a brief description of some popular medical terms and technologies mentioned in the reviews. Appendix B provides a brief description and explanation of how some common AI tools provide assistance in health informatics. Appendix C and Appendix D present more details about the reviews listed in Table 1 and Table 2, respectively.

## 2. Methods

Objective: The focus of this study is to synthesize the common concerns and opportunities raised by recent popular reviews on AI in health informatics.

Our literature search was conducted on 30 June 2025 through the popular academic database Scopus. The reason for choosing Scopus was that it is a comprehensive database of over one hundred million high-impact records, covering well a broad array of topics on both computer science and healthcare, which fits very well with the objective of our review. It consisted of the following two steps to select the recent popular reviews on AI and health informatics:

Literature Search I: The search was restricted to the Scopus terms “article title, abstract, and keywords”. The search was restricted to the combination “Artificial Intelligence” AND “Health Informatics”. The years covered were from 2023 onwards, and the article type was restricted to the type “review”. Another search was conducted with the restriction “Artificial Intelligence” AND “Health Analytics”.

Literature Search II: In this second stage, the focus is on the combination “Artificial Intelligence” AND “topic name”, where “topic name” refers to the popular topics of AI applications in health informatics. Here, the popular topics refer to the health informatics topics that have been frequently mentioned in the reviews, identified by analyzing the contexts of the above reviews by human experts. The goal of this stage was to select the highly cited reviews covering the popular topics for further analysis by human experts one by one. It was noted that, when setting the threshold at the 10 highly cited reviews for each topic, quite a number of highly cited reviews are excluded. On the other hand, when the threshold is set at values larger than 20, a few reviews that have only zero citations are included. Thus, the threshold of the 20 highly cited reviews was selected.

Note: It would be helpful to explicitly acknowledge that the limitations of this review include the citation lag, particularly given how quickly GenAI deployment and practice-based learning are evolving relative to citation cycles. Therefore, some interesting frontier GenAI deployments may not be included in this review, even though our supplementary literature search done on 20 January 2026 highlights our efforts to partly address this citation lag.

Supplementary Update I: a supplementary literature search was conducted on 20 January 2026, following the same procedure as the Literature Search I, in order to update the new literature data that have became available during the drafting of this review article.

Supplementary Update II: similarly, another supplementary literature search with the combination restriction using the terms “Artificial Intelligence” AND “Health Analytics” was conducted on the same day, i.e., 20 January 2026, following the same procedure as outlined in the above Literature Search II.

## 3. Results

### 3.1. Literature Search Results of AI and Health Informatics/Analytics

Literature Search I: For the combination “Artificial Intelligence” AND “Health Informatics”, a total of 40 reviews were found. For the combination “Artificial Intelligence” AND “Health Analytics”, a total of 6 reviews were found. Therefore, by combining these two searches together, a total of 46 reviews have been found. Key observations: It was found that 22 reviews covered clinical decision support, while the number of reviews covering patient care, electronic health records, hospital management, and remote patient monitoring were 16, 7, 7, and 7, respectively. It should be noted that there were reviews that covered several topics.

Literature Search II: For the five popular topics (clinical decision support, patient care, electronic health records, hospital management, and remote patient monitoring), a total of 100 influential review articles have been identified, and it was found that there were ten duplicate records. Note: The main reason for review article duplication is that a popular review may cover more than one topic. That review may appear as our selected review in multiple topic lists if it is popular across multiple topics. When we counted the total number of unique reviews that were covered in this review, we did not count this type of popular review multiple times. For the convenience of the practitioners, we make an effort to include these popular reviews in different topic lists. If a review that was popular across several topics was only included in one of its lists, some practitioners who were mainly interested in AI applications in one topic and who skipped other topic lists might have the chance of missing a few popular reviews on their specific target topic.

**Figure 1:**
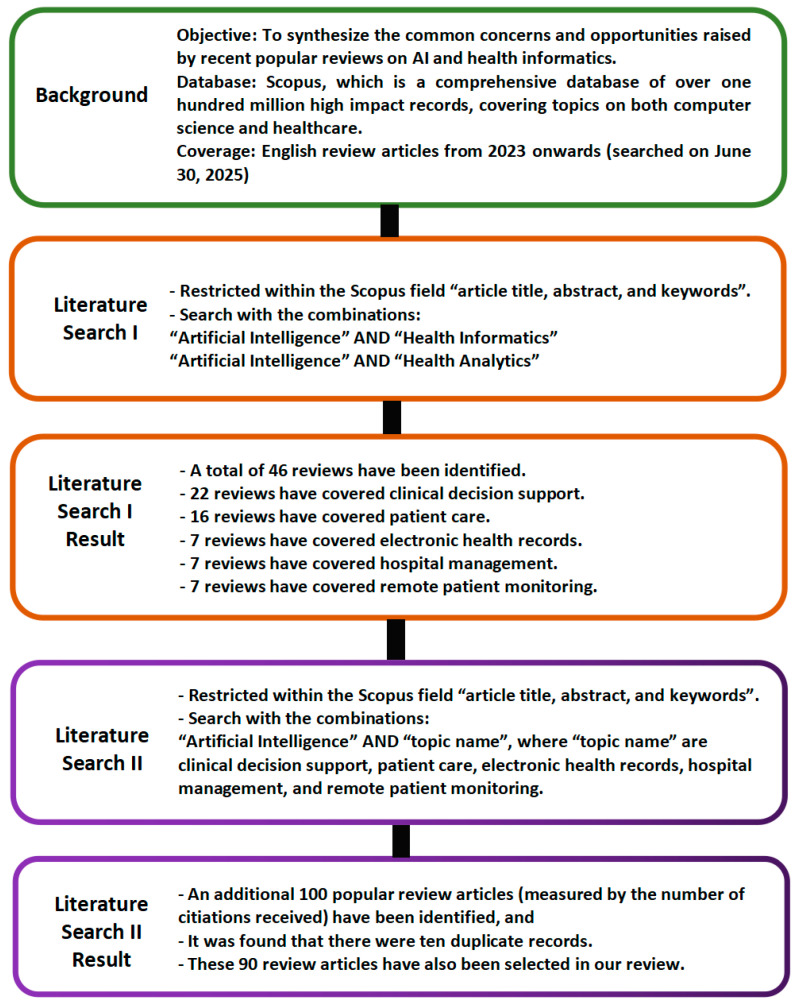
Framework of our Literature Search I and Literature Search II.

Thus, a total of 136 influential review articles have been identified after our Literature Searches I and II. All of these reviews are in English, and there are no further restrictions in the search results, besides the ones stated above. Figure 1 shows the framework of our Literature Search I and Literature Search II.

Supplementary Update I: A total of 89 review articles have been found (Note: Two Scopus search records were not included, as they were either only an overview of articles that were presented in a specific conference, or a column of articles in a particular journal). With the additional filter of the years covered set as 2023, 2024, and 2025, the total number of review articles has reduced to 54 reviews. In other words, there were only a total of 35 review articles that had been published before 2023, while fourteen new reviews have been published in 2025 after our literature search on 30 June 2025. For these fourteen new reviews, their main topics remain the same as the other 40 reviews in 2023, 2024, and 2025. Another observation is that in 2023, only 8 review articles were found. In 2024 and 2025, the number of review articles had increased rapidly to 24 reviews and 22 reviews, respectively. On the other hand, generally speaking, these 8 reviews in 2023 received much more citations than the reviews in 2024, while the reviews in 2024 have also obtained more citations than those published in 2025. The citation numbers of these eight reviews in 2023 are respectively: 136, 35, 20, 13, 12, 7, 3, and 0. The citation numbers of the twenty-four reviews in 2024 are respectively: 66, 50, 32, 29, 25, 22, 19, 16, 13, 11, 10, 6, 6, 6, 5, 5, 3, 3, 2, 1, 1, 1, 0, and 0. The citation numbers of the twenty-two reviews in 2025 are respectively: 7, 6, 5, 4, 3, 2, 2, 2, 2, 2, 1, 1, 1, 0, 0, 0, 0, 0, 0, 0, 0, and 0. This observation agrees with the fact that it takes time for other scientists to digest their findings and then cite their works.

Supplementary Update II: A total of 9 review articles have been found (Note: One Scopus search record was not included, as it is an editorial section of a specific journal). With the additional filter of the years covered set as 2023, 2024, and 2025, the total number of review articles has reduced to 7 reviews. In other words, there were only a total of 2 review articles that had been published before 2023, while one new review was published in 2025 after our literature search on 30 June 2025. In 2023, 0 review articles were found. In 2024 and 2025, the number of review articles had increased rapidly to 4 reviews and 3 reviews, respectively. On the other hand, generally speaking, the reviews in 2024 have obtained more citations than those published in 2025. The citation numbers of the four reviews in 2024 are respectively: 256, 63, 20, and 3. The citation numbers of the twenty-two reviews in 2025 are respectively: 3, 1, and 0.

**Figure 2:**
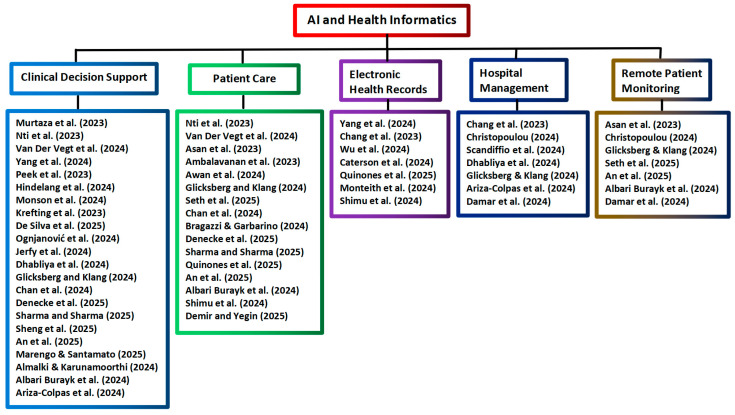
Tree diagram grouping the AI and health informatics applications from Scopus [1,4,13,14,15,16,17,18,19,20,21,22,23,24,25,26,27,28,29,30,31,32,33,34,35,36,37,38,39,40,41,42,43,44,45,46,47,48,49,50].

When comparing the contexts of the reviews from the literature search on 30 June 2025 and the reviews from the supplementary update on 20 January 2026, the main topics of these reviews remain the same, i.e., clinical decision support, patient care, electronic health records, hospital management, and remote patient monitoring. The analysis of the selected reviews on AI applications in health informatics is presented in Table 1, and the tree diagram for grouping the AI and health informatics applications selected from Scopus is shown in Figure 2.

**Table 1:** Highlights of the selected reviews on the recent AI applications in health informatics.

Authors	No. of Articles	Application Topics	Summaries
Murtaza et al. [13]	70	Clinical Decision Support	It explored various techniques for generating synthetic medical data with built-in privacy safeguards and evaluated different synthetic data approaches
Hua et al. [14]	31	Diagnostic Imaging	It identified the primary factors that influence how healthcare workers in medical imaging perceive and accept AI
Yang et al. [15]	8	Clinical Decision Support Genomic Medicine Electronic Health Records	Nine critical areas facing difficulties due to the rapid development of big data technologies in the context of precision medicine were identified
Nti et al. [1]	18,729	Clinical Decision Support Patient Care	The substantial number of publications reflects the increasing emphasis on leveraging technology to improve healthcare systems.
Van Der Vegt et al. [16]	37	Clinical Decision Support Patient Care	It focused on the use of artificial intelligence to predict real-time clinical deterioration in hospitalized adults
Asan et al. [17]	20	Patient Care Remote Patient Monitoring	It examined the influence of AI on consumer health informatics
Gurmessa and Jimma [18]	14	Diagnostic Imaging	It focused on the use of XAI in diagnosing breast cancer through mammography and ultrasound imaging
Yang et al. [19]	37	Clinical Decision Support Genomic Medicine	It centered on over-sampling strategies designed to manage multi-class imbalances in medical and other datasets, aiming to enhance the accuracy of AI-based predictive models.
Chang et al. [20]	24	Electronic Health Records Hospital Management	It explored the advantages of artificial intelligence, the Internet of Things, and personal health records in improving healthcare delivery
Wu et al. [21]	37	Electronic Health Records	It assessed burnout among healthcare professionals in relation to electronic health record usage
Christopoulou [22]	18	Remote Patient Monitoring Hospital Management	It investigated how ML is utilized in evidence-based telehealth and smart care
Peek et al. [23]	27	Clinical Decision Support	It highlighted that the shift toward digital health services is driven by AI and data sharing
Hindelang et al. [24]	18	Clinical Decision Support	It explored the role of chatbots in taking medical histories
Scandiffio et al. [25]	19	Hospital Management	It investigated how mentoring and coaching strategies support healthcare professionals in adopting digital tools, including artificial intelligence.
Caterson et al. [26]	76	Electronic Health Records	It focused on explainable AI applications in electronic health record analysis.
Ambalavanan et al. [27]	14	Patient Care	It explored how AI technologies can help understand long COVID
Monson et al. [28]	3939	Clinical Decision Support	It examined the use of AI in ophthalmology and vision science
Krefting et al. [29]	6	Clinical Decision Support	It examined current trends and applications of clinical decision support systems in obstructive sleep apnea management
De Silva et al. [30]	72	Clinical Decision Support	It explored how speech signal analysis can support the clinical decision process
Ognjanović et al. [31]	17	Clinical Decision Support	It examined developments in biomedical and health informatics
Awan et al. [32]	5	Patient Care	It explored the intersection of social media, crowdsourced data, and AI in public health
Jerfy et al. [33]	27	Clinical Decision Support	It highlighted the implementation of natural language processing in healthcare
Dhabliya et al. [34]	10	Hospital Management Clinical Decision Support	It explored the advantages of AI in the healthcare domain
Glicksberg and Klang [35]	50	Hospital Management Patient Care Remote Patient Monitoring Clinical Decision Support Genomic Medicine	It focused on the most impactful AI research in health and life sciences over the past three years
Seth et al. [36]	158	Remote Patient Monitoring Patient Care	It revealed prevalent topics within the Internet of Medical Things
Chan et al. [37]	33	Patient Care Clinical Decision Support	It focused on noninvasive blood glucose monitoring with artificial intelligence
Bragazzi and Garbarino [4]	8	Patient Care	It explored the emerging field of sleep infodemiology and infoveillance
Denecke et al. [38]	38	Patient Care Clinical Decision Support	It identified both enablers and barriers to adopting AI in participatory health informatics
Sharma and Sharma [39]	12,974	Patient Care Clinical Decision Support	It emphasized AI’s transformative potential to enhance patient outcomes, streamline healthcare operations, and spark innovation across the sector
Quinones et al. [40]	25	Patient Care Electronic Health Records	It explored how machine learning can predict patient-reported outcome measures following spinal surgery
Sheng et al. [41]	5	Clinical Decision Support Genomic Medicine	It focused on using AI for detecting oral cancer
Demir and Yegin [42]	32	Genomic Medicine Patient Care	It investigated AI’s role in disease prediction and genomic data analysis
An et al. [43]	867	Patient Care Remote Patient Monitoring Clinical Decision Support	It explored how artificial intelligence is being applied in obstructive sleep apnea
Marengo and Santamato [44]	63	Clinical Decision Support	It focused on the use of quantum computing to enhance AI applications in medicine and its role in strengthening the security of healthcare data
Almalki and Karunamoorthi [45]	17	Clinical Decision Support	Artificial intelligence is capable of contributing meaningfully to various phases of outbreak management
Albari Burayk et al. [46]	7	Patient Care Remote Patient Monitoring Clinical Decision Support	It investigated diabetic ketoacidosis and hyperosmolar hyperglycemic state, in addition to current clinical protocols and treatment procedures
Monteith et al. [47]	5	Electronic Health Records	It investigated the intersection of artificial intelligence, cybersecurity threats, and healthcare
Ariza-Colpas et al. [48]	1134	Hospital Management Clinical Decision Support	It examined the application of artificial intelligence in understanding long-term symptoms associated with COVID-19
Damar et al. [49]	32,046	Hospital Management Remote Patient Monitoring	It explored the influence of artificial intelligence, machine learning, robotic process automation, and virtual reality on healthcare systems
Shimu et al. [50]	9	Electronic Health Records Patient Care	It explored the role of health informatics in addressing drug addiction

**Figure 3:**
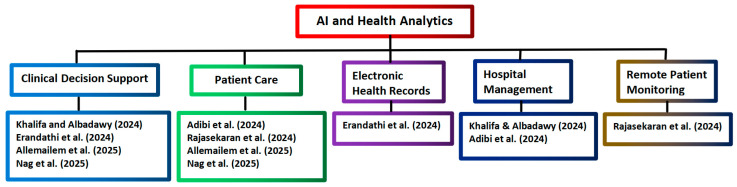
Tree diagram grouping the AI and health analytics applications from Scopus [5,52,53,54,55,56].

The above forty reviews in Table 1 focus on several main topics of health informatics. Nineteen of the above reviews focus on covering only one topic in each review, while in seventeen reviews, two topics are covered in each review. In three other reviews by Yang et al. [15], An et al. [43], and Albari Burayk et al. [46], three topics are covered in each review. There is only one review by Glicksberg and Klang [35] that covers five topics of AI applications in health informatics. The analysis of the selected reviews on AI applications in health analytics is presented in Table 2, and the tree diagram for grouping the AI and health analytics applications selected from Scopus is shown in Figure 3.

**Table 2:** Highlights of the selected reviews on the recent AI applications in health analytics.

Authors	No. of Articles	Application Topics	Summaries
Khalifa and Albadawy [5]	30	Hospital Management Diagnostic Imaging Clinical Decision Support	It explored how artificial intelligence is reshaping diagnostic imaging [51] in healthcare
Adibi et al. [52]	8	Hospital Management Patient Care	It reviewed how AI contributes to healthcare in smart environments where the spaces were equipped with sensors, digital tools, and location-aware services
Rajasekaran et al. [53]	11	Remote Patient Monitoring Patient Care	It focused on wireless body area networks topics, like protocols, security, authentication, and embedding with AI
Erandathi et al. [54]	102	Electronic Health Records Clinical Decision Support	It focused on using artificial intelligence to predict complications associated with diabetes mellitus
Allemailem et al. [55]	15	Clinical Decision Support Patient Care	It examined the role of multi-sensor technologies in the regular surveillance of patient health
Nag et al. [56]	123	Patient Care Clinical Decision Support	It explored how AI technologies are reshaping patient care through the lens of emotional intelligence and person-centered interventions

The above six reviews in Table 2 focus on several main topics of health analytics. In five reviews, two topics are covered in each review. In another review by Khalifa and Albadawy [5], three topics (hospital management, diagnostic imaging, and clinical decision support) are covered.

### 3.2. Recent AI Applications in Clinical Decision Support

The goal for decision support in healthcare is to integrate sophisticated computational techniques that replicate expert medical reasoning. These systems should aim not only to emulate clinical expertise but also to elevate the overall standard of healthcare delivery [57]. In clinical decision-making, AI enhances complex diagnostic and therapeutic procedures by seamlessly integrating with existing healthcare systems to deliver a more holistic understanding of a patient’s condition. Clinical decision support tools powered by AI contribute to better patient outcomes by analyzing large-scale data, uncovering hidden patterns, and delivering evidence-based guidance. AI-based clinical decision support can also help minimize false alarms and ease the burden on medical personnel [58]. These AI models have also shown strong performance in risk stratification and forecasting cardiac events [59]. The majority (85%) reported improved clinical outcomes following implementation of clinical decision support systems according to Wingfield et al. [60]. The main tools, summaries, key concerns, and opportunities of the selected reviews of AI applications in clinical decision support are highlighted in Table 3.

Among the AI tools listed in Table 3, traditional machine learning methods are still very popular, especially for prediction purposes in works by Revilla-León et al. [63], Lambert et al. [65], Poweleit et al. [67], Gala et al. [59], Khalifa and Albadawy [5], Theodosiou and Read [71], Aminizadeh et al. [73], Sakagianni et al. [74], and Jeong et al. [75]. GenAI is gaining its popularity in health informatics in the studies of Eggmann et al. [61], Chen and Esmaeilzadeh [62], Bhayana [64], Cascella et al. [8], Akinci D’Antonoli et al. [69], and Theodosiou and Read [71].

### 3.3. Recent AI Applications in Patient Care

Through the integration of patient-specific data, including medical histories and personal traits, AI technologies offer targeted insights that support the design of customized treatment strategies [5]. The use of AI and big data analytics have bolstered capabilities in forecasting disease outbreaks and tailoring medical interventions to individual patients, resulting in a 22% drop in repeat hospitalization [78]. By taking over repetitive administrative and clinical jobs, AI can free up time for clinicians to engage more meaningfully with patients, enhancing care quality [79]. This kind of proactive patient engagement can lead to improved outcomes and reduced healthcare costs, according to Davenport and Kalakota [80]. These intelligent models also support remote care and ensure patients stick to their treatment regimens [81]. AI also has high potential in aiding the care of patients with long-term illness and individual-focused care, which emphasizes aligning medical decisions with each patient’s individual inclination, beliefs, and requirements [82]. The main tools, summaries, key concerns, and opportunities of the selected reviews of AI applications in patient care are highlighted in Table 4.

Among the AI tools listed in Table 4, traditional machine learning methods are still very popular, especially for analysis purposes in studies by Vora et al. [81], Muteeb et al. [3], Maleki Varnosfaderani and Forouzanfar [85], Kufel et al. [87], Bekbolatova et al. [89], Krishnan et al. [9], O’Connor et al. [92], Marques et al. [94], and Hughes et al. [96]. GenAI is gaining its popularity in health informatics in the studies by Alowais et al. [83], Al Kuwaiti [84], Maleki Varnosfaderani and Forouzanfar [85], Safranek et al. [93], and Garg et al. [97].

### 3.4. Recent AI Applications in Electronic Health Records

An electronic health record (EHR) is a digital alternative to traditional paper medical files. It captures a wide array of patient information, including past diagnoses, test results, medications, vaccination records, and treatment plans [99]. These records typically contain both structured elements like numerical lab results and unstructured elements like clinical notes, with EHR unstructured data being the dominant data source according to Hossain et al. [100]. The adoption of EHR systems marks a transformative shift in healthcare information management, offering substantial improvements in patient care, decision-making, and managerial effectiveness [101]. Over the last decade and a half, the most significant technological shift in healthcare has been the widespread adoption of EHRs [102]. In 2009, only a small fraction of U.S. hospitals had implemented EHRs; by 2019, they had become nearly universal according to the Office of the National Coordinator for Health Information Technology [103].

Popular AI applications in EHR include tasks like predictive analytics and classification. Traditional rule-based systems often fall short in precision when compared to machine learning algorithms, according to Davenport et al. [80]. The rich, high-dimensional nature of EHR data, which may not be handled well by rule-based systems, enables deep exploration by advanced AI tools like deep learning and the discovery of previously unrecognized trends. Deep learning transformers can be deployed to provide a tailored solution for EHR data [104]. This AI architecture can effectively capture patient histories using various contextual embeddings such as age, timeline position, visit frequency, and event type. AI systems can analyze a variety of data in EHRs to detect early indicators of health conditions, and LLMs can automate the identification of HER reports to improve the timeliness of necessary subsequent procedures [69]. As a result, AI systems can support faster intervention and potentially better long-term outcomes. Because of AI’s high potential, giant technology corporations like Amazon were developing a novel AI tool to extract meaningful insights from unstructured EHR entries and clinical documentation [105]. The main tools, summaries, key concerns, and opportunities of the selected reviews of AI applications in electronic health records are highlighted in Table 5.

Among the AI tools listed in Table 5, traditional machine learning methods are still very popular, especially for information processing purposes in the works of Al Kuwaiti et al. [84], Hossain et al. [100], Zhang et al. [108], Wang and Wang [109], Marques et al. [94], Chakraborty et al. [112], Gupta and Kumar [115], Ullah et al. [116], Armoundas et al. [117], Gupta et al. [118], Knevel and Liao [119], Thakkar et al. [120], and Jimma [121]. GenAI is gaining its popularity in health informatics in the studies of Al Kuwaiti et al. [84], Harrer [107], Akinci D’Antonoli et al. [69], Chakraborty et al. [112], and Wachter and Brynjolfsson [113].

### 3.5. Recent AI Applications in Hospital Management

AI is able to contribute to improvements in hospital administration in many ways, while effective management of staff, equipment, and operating room scheduling is essential for high-quality surgical care [122]. AI technologies are instrumental in forecasting surgery durations, managing room availability, and anticipating cancellations [123]. Automated systems handle tasks like medical coding, invoicing, and scheduling, which not only cut costs but also reduce staff workload, enabling them to devote more time to direct patient care [124,125]. Process automation has lightened the managerial burden in healthcare facilities by 40% in the study by Baskar et al. [126]. AI-powered computerization in management is able to shorten waiting times by approximately 30%, thereby enhancing hospital throughput [127]. AI tools like virtual assistants and chatbots, which can work around the clock, help streamline services ranging from clinical documentation and appointment scheduling to personalized patient care [128,129]. AI translation tools and speech recognition systems also break down communication barriers [130,131]. The main tools, summaries, key concerns, and opportunities of the selected reviews of AI applications in hospital management are highlighted in Table 6.

Among the AI tools listed in Table 6, traditional machine learning methods are still very popular, especially for management supporting purposes in the works of Srivani et al. [132], Zuhair et al. [133], Almotairi [134], MacMath et al. [135], Gazerani [136], Bellini et al. [123], Hashemi et al. [137], Mumtaz et al. [138], Vargas et al. [140], Wingfield et al. [60], Tamir-Degabli et al. [144], Sánchez Suárez et al. [145], Green and Castro [146], and Auza-Santivañez et al. [149]. GenAI is gaining its popularity in health informatics in the studies of Bellanda et al. [139], Sánchez Suárez et al. [145], Green and Castro [146], and Kim et al. [147].

### 3.6. Recent AI Applications in Remote Patient Monitoring

AI plays a key role in enhancing the ecosystem of remote patient monitoring (RPM) systems, which are commonly employed to track the health of patients from a distance, including those receiving in-home care or hospitalized individuals. AI-assisted RPM systems have significantly advanced the delivery of care by enabling continuous, tailored data monitoring and processing in real time, according to Patel et al. [150]. AI-powered remote monitoring has the potential to lower re-hospitalization rates and improve chronic disease management. The sensor data from RPM need advanced AI systems to process and analyze in real time [151]. Both traditional and deep learning methods can be deployed to analyze vital signs and categorize physical activity, like heart rate, blood pressure, respiratory rate, and oxygen saturation. The proactive capability of AI tools for predictive analytics and clinical decision-making can help prevent health deterioration and optimize care [152]. The main tools, summaries, key concerns, and opportunities of the selected reviews of AI applications in remote patient monitoring are highlighted in Table 7.

Among the AI tools listed in Table 7, traditional machine learning methods are still very popular, especially for decision-making supports in the studies of Shaik et al. [153], Carini and Seyhan et al. [154]; Nashwan et al. [155]; Akinola and Telukdarie [157]; Liang et al. [158], Jayousi et al. [159], Park et al. [160], Patel et al. [150], Hamid et al. [161], Jin et al. [162], Olawade et al. [164], Umer et al. [165], Kraman et al. [166], Rabiee [168], and Glyde et al. [169]. GenAI is gaining its popularity in health informatics in the study of Umer et al. [165].

## 4. Discussion About the Common Concerns Synthesised from Reviews, and Future Directions

The objective of this section is to discuss the common concerns raised by the selected reviews and to highlight some future directions, as the advancement of AI in health informatics comes with many limitations. Major concerns include patient privacy, cybersecurity, ethics, clinical accountability, engaging health professionals, benchmarks and standardization, and lack of explainability.

**Concerns about patient privacy**: Privacy threats stem from adversarial efforts to extract confidential data, leading to potential information breaches according to Qayyum et al. [170]. Privacy concerns related to AI can be broken down into three key phases: (I) Data Collection: risks include tampered input devices, falsified data, and breaches of data regulations; (II) Training: threats involve adversarial inputs and compromised training datasets leading to flawed models; and (III) Deployment: issues include the spread of misinformation or hallucinated outputs and exploitation through adversarial attacks. Because AI tools are trained on vast and often sensitive patient data, they can be vulnerable to misuse and data breaches [171]. Specific concerns involve unauthorized identification, biometric data misuse, and unintended AI-derived inferences about sensitive health conditions. Protecting privacy is hampered by challenges such as maintaining scalability, interpretability, data authenticity, system robustness, and achieving a balance between data utility and confidentiality [106]. Despite these difficulties, it is necessary to ensure that actions are taken for compliance with data privacy regulations like GDPR and HIPAA, according to Baskar et al. [126]. A differential privacy policy is helpful for protecting sensitive health data while still allowing for practical usage.

**Concerns about cybersecurity**: Hospitals using AI and IoT have reported a 32% spike in cybersecurity incidents, underscoring the need for advanced encoding and secure verification protocols [172]. Protecting patient information may need the deployment of advanced techniques such as data encryption and decentralized data management. With the recent advances of LLMs in health informatics, this introduces new concerns related to patient privacy and cybersecurity that must be actively addressed [61]. Strict regulations for protecting patient data are needed, as chatbots often process highly sensitive health information and must be safeguarded against unauthorized access or data breaches.

**Concerns about ethics**: Glicksberg and Klang [35] emphasized that the speed of developing robust regulation should match the rapid evolution of new AI technologies, while Chan et al. [37] emphasized the need of addressing regulatory concerns in future AI-powered monitoring technologies. Even though there is a growing movement to define ethical frameworks for AI use in healthcare, much broader regulatory cooperation will be essential to tackle complex legal questions involving liability and intellectual property. Ethical standards must guide all innovations in healthcare technologies. Principles like equity, transparency, and respect for patient rights should guide decision-making processes, especially in critical care. Actionable regulatory structures must evolve continuously, while ethics must be foundational to any regulatory framework governing AI in health informatics. Organizations may individually propose ethnic guidelines. As an example, the American Medical Association [173] has initiated a set of principles to guide the design, deployment, and application of AI technologies. Nevertheless, efforts by an individual organization may only generate a limited impact. Broad cooperation is needed to promote principles that emphasize the importance of transparency, equity, and responsibility in AI development and use. Mechanisms should be set up for ongoing oversight and system updates to ensure AI remains safe, ethical, and clinically effective after deployment. Woodman and Mangoni [77] and Pinto-Coelho [86] suggested that the development of explainable systems is worthy for future AI systems, such that the regulatory expectations in healthcare may be satisfied.

**Concerns about clinical accountability**: AI models may reinforce or introduce biases, potentially compromising diagnostic accuracy and the fairness of treatment recommendations [174]. Even though GenAI has raised the algorithmic capability of the AI tools, the probabilistic, non-deterministic outputs of this paradigm challenge traditional notions of validation, auditability, and clinical accountability. Health practitioners face challenges that the probabilistic, non-deterministic outputs from this paradigm differ significantly from the traditional notions of validation, auditability, and clinical accountability in the healthcare industry. Sauerbrei et al. [90] emphasized that special efforts are needed to ensure AI systems are transparent, unbiased, and accountable. Building trust in AI systems depends heavily on ensuring transparency in how decisions are made and establishing clear lines of accountability among developers, institutions, and practitioners. In addition, it is crucial to identify and mitigate biases in AI models that could exacerbate healthcare disparities. Algorithmic bias may be actively mitigated through inclusive datasets and continuous validation.

**Concerns about engaging health professionals**: Beyond the efficiency gains and reshaping patient care through AI and person-centered interventions, AI and GenAI are reshaping skills, roles, and trust calibration among healthcare professionals. As the healthcare professionals have generally been slow to integrate them into clinical workflows [175], it is necessary that healthcare organizations build trust in AI among professionals. Lambert et al. [65] stressed the importance of involving healthcare professionals early in AI development and emphasized the need for tailored training programs to ensure smooth adoption in clinical environments. It is important to ensure consistent AI literacy among health practitioners [176] by developing actionable plans of tailored AI training programs for the medical staff members. According to Khalifa and Albadawy [5], there should be enough future initiatives focusing on developing ethical standards, equipping healthcare workers with appropriate training, and ensuring that AI advancements remain focused on patient needs. Many case studies have already focused on showing excellent model performance and technical capability for even the traditional machine learning tools in various medical applications. Here, an interesting perspective is highlighted, which is about the difference between algorithmic capability and capability-in-use. In real-world healthcare practice, value is often determined by integration into clinical workflows, organizational readiness, and governance. As outlined in studies like Liang et al. [158] and Bignami et al. [151], the need for advanced infrastructure and staff training still remains a major limitation. Therefore, there exists a need to focus more on the real-world implementation of how to effectively incorporate AI into pre-surgical planning, clinical workflows, etc.

**Concerns about benchmarks and standardization**: The absence of standardized outcome metrics is a main hindrance to clinical decision support systems [177]. In order to fully harness the potential of biomedical big data, it is essential to effectively tackle a range of challenges related to data quality, algorithm efficiency, security protocols, and standardization across systems [15]. In response to the growing need for robust evaluation of AI tools, more efforts and actions should be put into the development of new frameworks and benchmarks for AI tools. It is encouraging to notice that new reporting frameworks such as STARD-AI, PROBAST-AI, SPIRIT-AI, CONSORT-AI, and DECIDE-AI have emerged. The DECIDE-AI guideline, in particular, addresses the evaluation of ML tools in early-stage, small-scale clinical settings, according to Vasey et al. [178].

**Concerns about lack of explainability**: The deployment of explainable AI may address the concerns about the lack of explainability in AI applications and has been gaining popularity in health informatics applications. Caterson et al. [26] focused on explainable AI applications in electronic health record analysis. Hulsen [72] talked about the need to make AI more explainable in future applications. Woodman and Mangoni [77] raised the need to develop explainable ML approaches that align with ethical standards and regulatory expectations in healthcare. Nevertheless, revealing how an AI model arrives at decisions could inadvertently expose sensitive patient data or enable malicious manipulation through reverse engineering [179]. The use of XAI introduces challenges related to data privacy and system security [72].

One future direction is to focus on how to fully utilize these new AI paradigms in healthcare with synthetic data by generative AI. Synthetic data presents a promising route to overcoming the privacy issues often associated with using real patient information in AI applications, according to Murtaza et al. [13]. With the recent advances in generative AI technologies, synthetic data is increasingly seen as a privacy-compliant alternative to real datasets and is gaining traction in healthcare communities. Chen et al. [180] emphasized that synthetic data plays a valuable role in evaluating novel artificial intelligence algorithms.

Another future development may focus on enhancing chatbot models with advanced AI for history-taking and incorporating them into clinical decision support systems for real-time, evidence-based recommendations [24]. Despite its capabilities, ChatGPT has notable flaws, such as factual inaccuracies, nonsensical content, and an inability to reliably provide medical advice. Given these limitations, ChatGPT is currently unsuitable for decision-making purposes in clinical environments [181]. Additionally, despite their utility, current chatbot systems still fall short in replicating the empathy and psychological understanding that human beings naturally offer, according to Li et al. [182].

The third future direction is about how to better implement/embed AI tools like LLMs for tasks related to electronic health records and hospital management in real-world healthcare environments. For example, AI can enhance evidence-based medical imaging selection. In documentation, AI can be deployed to automatically generate and update medical records by integrating data from various systems, reducing manual entry errors and enabling better-informed clinical decisions [183]. In inventory management, predictive AI models help hospitals maintain optimal supply levels by analyzing usage patterns and external factors like seasonal illnesses [184,185]. Financially, AI supports hospitals by automating tasks such as revenue forecasting, invoice auditing, and financial reporting, contributing to more strategic resource planning [186,187]. Deeper embedding of AI technologies into real-world settings may lead to even better healthcare operations.

The fourth future direction is to deploy more AI paradigms for healthcare, for example, continual learning, transfer learning, and few-shot learning, which are gaining traction from practitioners [188]. These advanced AI tools can allow effective model development, even with limited datasets. It became well-known that predictive models trained within a single health system often perform poorly when applied to new settings or populations, limiting their transferability, according to Wiens et al. [189]. On the other hand, some earlier studies show that advanced AI tools like transfer learning and few-shot learning could overcome these kinds of issues and successfully brought important advancements for AMR research [190].

The fifth future direction is about how to apply AI with social media data for better healthcare. AI has the potential to provide essential processing for social media data, according to Adishesha et al. [191], while social media content offers rich data that can inform various applications, including healthcare. There are opportunities for AI applications in sentence prediction and user-generated tagging. For example, AI-powered content analysis could provide a better understanding of public sentiment, opinions, and attitudes, particularly regarding digital education, during the COVID-19 pandemic [192].

The sixth future direction is to promote more multinational efforts of AI initiatives in health informatics in developing countries. As noted by Jimma [121], most AI research outputs to date have originated from wealthier nations, despite the fact that the majority of the world’s population lives in low- and middle-income countries. According to the 2021 Stanford AI Index, East Asia led AI-related academic publications with 26.7% of the total, followed by the United States with 14% [193]. This imbalance stems from disparities in research funding, infrastructure, institutional priorities, capacity, and even language barriers, according to Reis et al. [194]. More multinational efforts of AI initiatives for developing countries may help improve this imbalance. For example, AI-powered RPM is making psychiatric care more accessible in remote and underserved areas [195]. Through encrypted video consultations, psychiatrists can remotely assess and treat patients, ensuring consistent care and enabling collaboration with local health providers to deliver more holistic support [196,197]. Similarly, AI tools enable self-monitoring and remote diagnostics in eye care [162]. Better augmented reality and virtual reality technologies may further enhance remote consultations, according to Kouijzer et al. [198].

## 5. Conclusions

Sustainable healthcare represents a comprehensive approach to healthcare delivery that incorporates not only clinical outcomes but also economic, social, and environmental factors. The rapid advancement of technologies like artificial intelligence, the Internet of Things, big data analytics, and cloud computing are playing a crucial role in promoting the development of sustainable healthcare, according to Pammi et al. [199]. AI models contribute to healthcare sustainability by improving overall efficiency in healthcare [200], which in turn supports long-term organization sustainability.

A major future vision of AI in health informatics is to develop AI-assisted tools, like reliable virtual clinical assistants, to enable sustainable healthcare. These AI-powered virtual clinical assistants should be capable of deepening patient engagement, providing individualized health guidance, and supporting long-term stewardship of chronic illnesses [8]. It would also be interesting to explore the combination of blockchain technologies in the deployment of AI tools, as highlighted by Zhao et al. [201]. As shown by Qi et al. [202], traditional machine learning may not be able to provide desirable performance when handling complex issues. Applications of contrastive learning [203] may be very helpful for some difficult health informatics issues. While developing any of these AI projects in healthcare, collaborations among different stakeholders are needed to tackle the key concerns identified in this review for achieving better AI-assisted sustainable healthcare.

## Figures and Tables

**Table 3:** Highlights of the selected reviews on the AI applications in clinical decision support.

Authors	Main Tools	Summaries	Key Concerns and Opportunities
Eggmann et al. [61]	LLMs ChatGPT	Application of ChatGPT and other large language models in dental medicine	- Concerns related to patient privacy and cybersecurity must be addressed - Robust quality assurance processes are needed to evaluate health-related outputs generated by LLMs
Chen and Esmaeilzadeh [62]	Generative AI	Using generative AI in healthcare	- Vulnerable to misuse - Crucial to address associated risks to ensure safe deployment
Revilla-León et al. [63]	Expert systems Case-based reasoning Neural Network	AI applications in diagnostic aid in dentistry	Further research is needed to validate its clinical effectiveness
Bhayana [64]	LLMs Chatbots	LLMs applications in radiology	- Limitations included hallucinations, lack of deep reasoning, data security concerns, and high computational demands - Future research should aim to develop solutions that specifically target and address these issues
Lambert et al. [65]	Machine learning	Factors that affect healthcare professionals’ adoption of AI	- There existed fears around diminished clinical autonomy and challenges in incorporating AI into routine clinical processes - Tailored training programs are needed to ensure smooth adoption in clinical environments
Ramgopal et al. [66]	AI Big data NLP-based tool	AI-powered clinical decision support in pediatric healthcare	- AI’s use in pediatric settings remains limited - Further research was needed to unlock the full potential of AI in this area of care
Poweleit et al. [67]	Machine learning XGBoost model Deep learning	AI in therapeutic drug monitoring and precision dosing	- Findings indicated that AI can effectively support initiatives in drug - Interdisciplinary collaboration is needed
Muteeb et al. [3]	AI	AI on combating antibiotic resistance	Key vulnerabilities were identified, particularly algorithmic bias and concerns regarding data privacy, and ethnics [68]
Cascella et al. [8]	NLP LLMs Chatbots	Large language models in the medical field	Future vision involves the transformation into reliable virtual clinical assistants that are capable of deepening patient engagement
Gala et al. [59]	Machine learning Predictive AI Neural networks	Integration of AI in cardiology	- High costs and challenges in deployment remain major barriers to adoption - Future innovation may center on fully leveraging AI to enhance prevention and real-time monitoring
Akinci D’Antonoli et al. [69]	LLMs NLP Deep learning ChatGPT	ChatGPT, prompt engineering, and their use in healthcare and radiology	- Prone to producing misleading or entirely fabricated content - To prioritize the development of privacy-preserving AI technologies that can operate safely and responsibly
Vimbi et al. [70]	XAI	XAI for the diagnosis of Alzheimer’s disease	Future research could apply these interpretability techniques to improve the trustworthiness and clinical value of AI-powered decision support tools
Khalifa and Albadawy [5]	Machine learning Deep learning CNN	AI in diagnostic imaging	To focus on implementing ethical standards and providing appropriate training for healthcare professionals to ensure responsible use
Theodosiou and Read [71]	Machine learning Deep learning Neural network ChatGPT	AI in clinical infection management	To address ethical concerns, particularly regarding algorithmic bias and the lack of transparency
Hulsen [72]	XAI Granular computing Fuzzy modeling	Explainable AI in healthcare by promoting greater clarity and user trust	- The use of XAI may introduce challenges related to data privacy and system security - Future efforts to make AI more explainable must not come at the expense of protecting patient confidentiality
Aminizadeh et al. [73]	Machine learning Deep learning CNN	AI and distributed computing applications in healthcare	There is a need to address security challenges and create stronger mechanisms to safeguard sensitive health data in increasingly interconnected systems
Sakagianni et al. [74]	Machine learning XGBoost	AI in predicting antimicrobial resistance	Techniques such as transfer learning and few-shot learning are gaining traction, as they allow for effective model development even with limited datasets
Jeong et al. [75]	Machine learning CNN	Deep learning in dermatology	- AI tools are valuable diagnostic aids for skin conditions - AI models enhance diagnostic precision and lower workload and costs
Fitzpatrick [76]	Deep learning	AI’s performance in clinical decision support	- Presence of biases in training datasets remains a concern - Future research may need to focus on enhancing the transparency of AI models
Woodman and Mangoni [77]	Machine learning Reinforcement learning	AI applications in geriatric medicine	- Existing limitations include algorithmic bias, lack of transparency, and regulatory challenges - To develop explainable ML approaches that align with ethical standards and regulatory expectations in healthcare

**Table 4:** Highlights of the selected reviews on the AI applications in patient care.

Authors	Main Tools	Summaries	Key Concerns and Opportunities
Alowais et al. [83]	NLP LLM CNN	AI in illness identification, personalized treatment planning, and enhancing patient interaction	- Concerns over data privacy, inherent biases, and the requirement for human oversight - To prioritize building AI systems that are both effective and ethically sound, while encouraging strong partnerships
Vora et al. [81]	Machine learning Deep learning NLP	AI applications in pharmaceutical fields	- Limitations include data scarcity, algorithmic bias, opacity in decision-making, ethical issues, and insufficient expert support - To refine AI for more efficient pharmaceutical logistics, manufacturing, and drug distribution
Muteeb et al. [3]	Machine learning	AI as a tool for antimicrobial resistance	- Bias in algorithms and patient privacy concerns remain significant barriers - To develop scalable AI models capable of rapidly analyzing huge amounts of data on shifts in resistance trends
Al Kuwaiti [84]	Machine learning NLP Transformer ChatGPT	AI applications in virtual monitoring, patient adherence, and therapeutic care	- Challenges include ethical considerations, affordability, data governance, informed consent, and equitable access - To develop AI systems that are socially responsible, technically sound, and aligned with patient-centered values
Maleki Varnosfaderani and Forouzanfar [85]	Machine learning Neural network Transformer Generative adversarial network	AI in clinical workflows, operations, scans, and patient monitoring	- Challenges include ethical concerns, data security, and bias in algorithms - To prioritize collaborative efforts across disciplines, encouraging dialog between healthcare professionals and researchers to address these implementation hurdles and ensure responsible integration of AI tools
Pinto-Coelho [86]	Deep learning CNN GAN	AI applications in radiology, pathology, cardiology, and patient care	- Persistent barriers include limited data access, lack of model transparency, and regulatory constraints - To make AI more interpretable, enabling clinicians to understand how diagnostic models reach their conclusions
Kufel et al. [87]	Machine learning Neural networks Deep learning	AI in improving patient outcomes	- Obstacles include insufficient high-quality training data, privacy concerns, and risks of cyberattacks - Overreliance on automated systems also raises the possibility of sidelining human clinical judgment - To emphasize responsible AI applications complementing medical expertise
Williamson and Prybutok [88]	Federated learning Ethical AI	AI in patient care	- Barriers include privacy issues, patient autonomy, and maintaining data integrity - To promote a holistic approach to design fair, effective, and responsible AI systems for healthcare
Bekbolatova et al. [89]	Machine learning Neural network NLP	To enhance patient care and streamline healthcare services with AI	- There are notable legal and ethical obstacles - Future efforts should include widespread public education to build trust and foster acceptance of AI
Chen and Esmaeilzadeh [62]	Generative model GAN LLM	Impact of generative AI on clinical diagnostics, therapy design, and healthcare management	- Serious concerns around data security, privacy, and ethical considerations - To ensure responsible use of generative AI with targeted strategies to safeguard sensitive information and promote transparency
Krishnan et al. [9]	Machine learning Nural network Deep learning	AI in history-taking, physical checking, scan analysis, treatment planning, and prognosis prediction	- Key barriers include ethical, legal, and societal concerns, especially around public trust and data protection - Future approaches should emphasize collaboration between human clinicians and AI systems to produce better outcomes
Sauerbrei et al. [90]	Explainable AI	How AI affects sympathy and kindness in patient care	- Major challenges include ensuring AI systems are transparent, unbiased, and accountable - To develop AI tools that meet various standards
Elendu et al. [91]	Artificial intelligence	Ethical implications of incorporating AI and robotics into patient care	- Main concerns are data privacy and cybersecurity for applications in telemedicine, robotic surgery, and customized recovery - To foster more seamless collaboration between AI tools and clinicians
O’Connor et al. [92]	Machine learning Neural network NLP	AI applications in nursing and midwifery	- Challenges include low-quality datasets, bias, limited clinical interpretability, concerns around privacy and trust, and a lack of AI literacy among healthcare practitioners - To focus on robust digital health datasets and multidisciplinary research
Safranek et al. [93]	LLM ChatGPT	Roles of LLMs like ChatGPT in medical education	- Concerns that the internal logic of AI may not align with the goals of human-centered medical practice - To address AI hallucinations, inherent biases, uncertainty in model outputs, and associated legal and ethical risks
Marques et al. [94]	Machine learning XGBoost Interpretable AI	AI roles in silico methods for advancing precision medicine	- Key challenges include inconsistent data quality in pharmacometrics and the absence of unified standards - To embed machine learning into pharmacometric models to improve dosing precision
Chen et al. [95]	Machine learning Random forest CNN Federated learning	AI-assisted precision medicine in lung cancer treatment	- The main challenge is the risk of data breaches - To adopt federated learning approaches, which allow institutions to collaboratively develop models without sharing patient-level data
Hughes et al. [96]	Machine learning Deep learning Reinforcement learning	AI-enhanced wearables for early detection and management of cardiovascular diseases	- Key obstacles remain in privacy protection and data security - To foster collaboration between healthcare professionals and AI specialists to establish common standards
Garg et al. [97]	ChatGPT NLP LLM	ChatGPT applications in healthcare delivery and medical research	- Concerns persist regarding the originality of responses, data privacy, factual accuracy, embedded bias, and legal implications - To develop clear policy and ethical guidelines to ensure safe and effective deployment of ChatGPT
Cellina et al. [98]	Deep learning Neural network	The role of digital twins in healthcare	- AI is essential for managing and interpreting the vast datasets that fuel these models - Key challenges involve ensuring data integrity, secure handling, interoperability, and fairness - To develop standards with multi-stakeholder collaboration

**Table 5:** Highlights of the selected reviews on the AI applications in electronic health records.

Authors	Main Tools	Summaries	Key Concerns and Opportunities
Al Kuwaiti et al. [84]	Machine learning NLP Transformer ChatGPT	Integration of artificial intelligence in the healthcare	- Challenges, including ethical, social, and technical concerns - To ensure robust governance frameworks around AI deployment for patient safety and responsibility
Khalid et al. [106]	Federated learning	Privacy-preservation AI strategies in healthcare	- Concerns involve unauthorized identification, biometric data misuse, unintended AI-derived inferences about sensitive health conditions, and privacy - Federated learning and hybrid AI methods hold promise for advancing secure and ethical AI
Harrer [107]	Deep learning Generative AI LLM	LLM’s effect on healthcare data workflows	- Key ethical concerns surrounding LLMs include clearness, responsibility, justice, value alignment, data privacy, and interpretability - To include ethical design principles that support both AI and human judgment
Hossain et al. [100]	Machine learning Deep learning NLP	Roles of ML, deep learning, and NLP on EHR	- Challenges include selecting appropriate ML models and handling data imbalances - To develop systems that address these challenges
Zhang et al. [108]	Machine learning CS-SVM Deep learning	How artificial intelligence is applied in cancer prediction	- Challenges include safeguarding patient privacy, integrating split datasets, scaling AI systems, and limited interpretability - To extend beyond oncology to enhance diagnosis, prognosis, and patient well-being
Wang and Wang [109]	Machine learning Deep learning	AI in precision medicine	- To develop more sophisticated AI models capable of processing large, diverse datasets for personalized care strategies
Marques et al. [94]	Machine learning XGBoost Interpretable AI	AI-driven computational approaches in advancing precision healthcare	- Concerns include ethical implications [110], data protection, and supervisory compliance - To prioritize interpretable AI models to ensure transparency and trust in clinical applications
Murtaza et al. [13]	GAN	Producing privacy-preserving synthetic medical data	- Limitations, such as the lack of longitudinal datasets and the absence of standardization [111] and benchmarks - To combine synthetic health record models with hybrid data generation strategies
Akinci D’Antonoli et al. [69]	Deep learning LLM ChatGPT NLP	LLMs in radiology	- LLMs automate the identification of reports that suggest additional imaging - Facing ethical and legal barriers - To advance LLM applications in medical imaging
Chakraborty et al. [112]	Machine learning Deep learning Chatbot ChatGPT	Data-driven AI in medicine	- Barriers include inconsistent data formats, unclear data policies, workforce shortages, ethical dilemmas, and cybersecurity risks - Interdisciplinary collaboration is needed to develop AI algorithms to enhance diagnostic capabilities
Wachter and Brynjolfsson [113]	GenAI ChatGPT	Generative AI applications in healthcare	- Issues include hallucinations, algorithmic bias, safety concerns, and cost-effectiveness - To develop APIs and peripheral infrastructure for smoother integration between genAI and EHR platforms
Fitzpatrick [76]	Deep learning	Digital communication tools in enhancing health outcomes	- Concerns included data security and patient privacy [114] - To mitigate bias and avoid misunderstandings in AI-driven clinical decisions
Gupta and Kumar [115]	Machine learning Deep learning	AI applications on health datasets for tailoring patient treatments	- A significant challenge is the fragmentation of healthcare systems - To enhance neurological care through more sophisticated AI applications
Ullah et al. [116]	Machine learning	Intelligent technologies in managing cardiovascular disease	- Issues include transparency and ethics - Future developments in AI for cardiovascular health must prioritize transparency and ethical standards to fully realize their clinical impact
Armoundas et al. [117]	Machine learning	How AI supports precision medicine in cardiovascular care	- Challenges include acquiring, curating, and securely sharing quality imaging data - To build cost-efficient, clinically applicable AI models designed to meet pressing medical needs
Gupta et al. [118]	Machine learning SVM KNN Deep learning	Applications of AI, Internet of Things, and EHRs in Parkinson’s disease	- It is challenging to achieve early and accurate diagnosis of the disease - To enhance the precision and reliability of AI diagnostic tools for Parkinson’s disease
Knevel and Liao [119]	Machine learning NLP	How machine learning techniques can extract meaningful, applicable patterns from EHRs	- EHR datasets suffer from limitations like inconsistencies - EHRs present unique challenges like access disparities, inherent noise, and biases due to care indications - To develop robust approaches to address these concerns
Thakkar et al. [120]	Machine learning Deep learning Reinforcement learning NLP	AI applications in mental healthcare	- Concerns include ethical dilemmas, data privacy, and accuracy and dependability of current models - To develop AI tools that are well-validated across diverse patients and that prioritize transparency, ethical integrity, and responsibility
Jimma [121]	Machine learning Deep learning NLP	AI use in healthcare	- Imbalance of AI research between developed and developing countries - To build sophisticated AI systems for early detection and proactive disease management

**Table 6:** Highlights of the selected reviews on the AI applications in hospital management.

Authors	Main Tools	Summaries	Key Concerns and Opportunities
Srivani et al. [132]	Machine learning NLP Cognitive analytics	Exploring the role of cognitive computing in healthcare	- Challenges include concerns over data security, lengthy implementation processes, and lack of full automation - To develop autonomous and adaptive systems capable of tackling complex healthcare issues
Zuhair et al. [133]	Machine learning Deep learning	AI applications in healthcare across developing nations	- Hindrances include ethical and transparency issues, concerns over fairness and bias, and poor infrastructure - To foster cross-disciplinary collaborations between healthcare providers and AI experts
Almotairi [134]	Machine learning	Smart healthcare with Internet of Medical Things	- Barriers include the generation of excessive data, data privacy and security, and high cost - To develop predictive systems for neurological events like strokes and seizures
MacMath et al. [135]	Machine learning Neural network	AI in clinical allergy	- Hindrances include regulatory hurdles, transparency, reproducibility issues, and legal and privacy concerns - To refine AI tools to better analyze allergic conditions, supporting more individualized approaches to care
Gazerani [136]	Machine learning Deep learning	Digital twin technology in managing neurological and other health-related conditions, using AI for advanced analysis	- Hindrances include the absence of standardized data structuring and flow, particularly in applications like migraine treatment - To address ongoing concerns around data security and confidentiality
Bellini et al. [123]	Machine learning XGBoost Random forest Neural network	How AI supports operating room management	- Concerns include data access and patient privacy - To focus on interprofessional collaboration to harness AI’s full potential for improving administrative efficiency
Hashemi et al. [137]	Machine learning Deep learning	AI in assisting anesthetic decision-making	- A significant limitation is the limited interpretability of many machine learning outputs - To develop transparent, user-friendly AI tools that can be readily adopted by anesthesiologists
Mumtaz et al. [138]	Machine learning Support vector machine Neural network	AI in advancing precision medicine	- Lack of harmonized data standards continues to introduce bias and privacy challenges - To refine data handling practices and unlock the capabilities of AI in precision medicine
Bellanda et al. [139]	LLM ChatGPT	Examining the role of ChatGPT in retinal care	- Concerns include accuracy, dependability, and risk of generating misinformation - To enhance the tool’s precision, ensuring alignment with current clinical regulations
Vargas et al. [140]	Machine learning Deep learning	Healthcare Maturity Models, and lessons learned during the COVID-19 pandemic	- Practical difficulties persist in deploying and managing such technologies effectively - To prioritize the integration and coordination of technologies, such as machine learning, deep learning, robotic systems, wearables, and IoT devices
Wahyuni et al. [141]	Artificial intelligence	AI innovation within hospital healthcare delivery	- Persistent concerns include data security, patient privacy, and equitable access to healthcare - To advocate among healthcare workers to ensure they can effectively adapt to evolving digital health tools
Yilmaz et al. [142]	Artificial intelligence	How AI is integrated into neurosurgical procedures	- Ethical oversight, cybersecurity, data ownership, and patient privacy remain critical concerns - To integrate AI solutions in ways that enhance both the protection and operational performance of neurosurgical practices
Hardavella et al. [143]	CNN	Lung cancer screening and the management of pulmonary nodules	- AI has strong potential for opportunistic screening applications - To prioritize the development of adaptive strategies to restructure healthcare services in a way that supports the growing demand for lung cancer filtering
Wingfield et al. [60]	Machine learning Neural network CNN	Clinical Decision Support Systems on patient outcomes	- A key challenge remains the absence of standardized outcome metrics - To embody AI into decision support systems to further enhance their effectiveness in clinical practice
Tamir-Degabli et al. [144]	AI Machine learning	How salvage therapy and patient-specific factors influence management strategies	- Application of AI and predictive modeling in acute severe ulcerative colitis remains largely experimental - To focus on using AI for patient risk classification to support more informed therapeutic judgments
Sánchez Suárez et al. [145]	Machine learning Deep learning CNN Chatbot	AI in improving hospital workflows	- AI systems enhance healthcare quality and free up staff to concentrate on more complex responsibilities - AI tools improve resource utilization and minimize errors, transforming traditional operations
Green and Castro [146]	Machine learning NLP Chatbot	AI applications in otolaryngology	- There is a rapid expansion of AI-driven tools aimed at enhancing operational efficiency - To further explore innovative AI applications in clinical workflow management
Kim et al. [147]	Chatbots ChatGPT	Integration of chatbots into dermatological care	- Main concerns are patient safety and data privacy - Future development of chatbots must prioritize patient safety, data privacy, and ensure that these tools supplement human clinicians
Krotkiewicz et al. [148]	Artificial intelligence	How medical institutions are evolving through the adoption of digital technologies like AI	- Obstacles include elevated cybersecurity threats, substantial initial investment requirements, and reluctance among healthcare professionals - To focus on AI-supported diagnostics and IoT-based automation
Auza-Santivañez et al. [149]	Machine learning Neural network	How AI is applied in intensive care units	- Challenges include AI system plainness, incorporation into clinical workflows, and ethical considerations - To prioritize the development of more interpretable AI tools for precision medicine

**Table 7:** Highlights of the selected reviews on the AI applications in remote patient monitoring.

Authors	Main Tools	Summaries	Key Concerns and Opportunities
Shaik et al. [153]	Machine learning Reinforcement learning Federated learning Explainable AI	AI in remote patient monitoring	- Limitations include poor interpretability, data privacy concerns, uncertainty in models and data, challenges in processing signals, class imbalances, insufficient data volume, and inconsistencies in patient information - To emphasize the integration of explainable AI, federated approaches, and reinforcement learning strategies
Carini and Seyhan et al. [154]	Machine learning Deep learning	AI-based tools to enhance remote monitoring	- Challenges include poor data quality, limited availability of annotated datasets, data security and privacy risks, algorithmic bias, regulatory and ethical constraints, and high installation costs - Ethical engagement of all stakeholders to ensure AI advancements do not worsen healthcare inequalities
Nashwan et al. [155]	Machine learning Deep learning NLP Support vector machine	AI’s potential to support RPM	- Key concerns revolve around protecting patient privacy and maintaining human interaction within the care process - To promote ethical AI deployment, preserving strong nurse–patient relationships, and improving telehealth platforms
Thacharodi et al. [156]	Deep learning NLP	AI’s transformative impact on patient diagnosis and care delivery	- Persistent issues like data security, accessibility, and equity - To advance telemonitoring tools that are scalable, efficient, and broadly usable - To standardize sensor technologies to help lower administrative burdens and improve adoption
Akinola and Telukdarie [157]	Machine learning NLP	Integrating AI within data analytics and patient-centered care	- Challenges include limited interconnectivity and concerns over data security - To address technology-related disparities and ensure these tools are effective even in low-resource situations
Liang et al. [158]	Machine learning	Leveraging AI to create predictive, individualized treatment strategies in remote care	- The need for advanced infrastructure and staff training remains a major limitation - To further incorporate AI into pre-surgical planning, clinical workflows, and remote digital health systems
Jayousi et al. [159]	Machine learning NLP	How information and communication technologies are reshaping nursing and patient care management.	- Smart healthcare platforms can offer individualized recommendations - Persistent challenges include data interconnectivity, security, and gaining user trust - Future initiatives should align with patient-centered care principles to enhance the care experience
Park et al. [160]	Machine learning Deep learning CNN-LSTM	AI-enabled video surveillance for the elderly	- Key challenges include maintaining video quality, complying with privacy regulations, and ensuring accurate training data - To prioritize ethical compliance, data protection, and adherence to governmental frameworks
Patel et al. [150]	Machine learning Neural network Deep learning	AI in remote patient monitoring to manage chronic conditions	- Challenges include data security, patient privacy, and managing the vast amounts of information generated - To refine AI-enhanced RPM systems to enable more targeted treatment strategies
Hamid et al. [161]	Machine learning Random forest Deep learning	Machine learning in diagnostic and clinical support	- Main concerns are around the algorithmic bias of the AI systems - To implement deep learning techniques to enhance decision support systems and ensure consistent AI literacy among clinicians
Jin et al. [162]	Machine learning Deep learning CNN	Smartphone-based AI tools in ophthalmology	- Obstacles include data protection, validation of models, regulatory compliance, and building public acceptance - To focus on cross-sector collaboration to responsibly address these ethical and technical concerns
Aldzhyan et al. [163]	Artificial intelligence	Impact of telemedicine on managing gastrointestinal disorders	- How AI can be fully harnessed in tele-gastroenterology remains uncertain - There is a need for further exploration and development to fully harness the potential of AI
Olawade et al. [164]	M achine learning Deep learning	AI and wearable technologies in stroke-related care, spanning prevention, diagnosis, and recovery	- Challenges include data precision, privacy issues, and the seamless incorporation of wearable devices into healthcare infrastructure - To prioritize resolving these limitations to optimize clinical integration
Umer et al. [165]	Machine learning Deep learning ChatGPT	AI in the caring of patients in Pakistan	- Ethical and operational concerns include patient privacy, algorithmic bias, ensuring explicitness, and maintaining human oversight in treatment decisions - To build trustworthy, secure AI systems and foster broad cooperation to guide ethical development
Kraman et al. [166]	Machine learning	How AI and remote monitoring technologies are deployed to evaluate respiratory health	- AI can identify acoustic patterns such as wheezing, coughing, or snoring - Future work should enhance AI’s ability to interpret respiratory sound and automate diagnostic classification
Erickson et al. [167]	Artificial intelligence	AI-driven virtual rehabilitation platforms	- Collected data is transmitted in real time to clinicians - To compare the effectiveness of remote recovery with traditional in-person therapy
Bignami et al. [151]	Artificial intelligence	How wearable devices contribute to early detection systems	- Barriers include funding, staff training, data protection, user consent, and technological reliability - To develop an AI-based clinical decision system to minimize false alarms and ease the burden
Rabiee [168]	Machine learning Ensemble machine learning Probabilistic AI Reinforcement learning	Wearable biosensors in healthcare	- AI plays a critical role in transforming the raw data into meaningful, personalized health insights - Concerns include biocompatibility of devices, long-term functionality, and scalable solutions - To prioritize cross-disciplinary AI development to predict health events, eliminate signal interference, and identify irregularities in biosignals
Glyde et al. [169]	Machine learning Neural network Bayesian network	How machine learning can be applied in remote monitoring systems to anticipate acute exacerbations in patients	- Main hindrances are inconsistent methodologies and insufficient datasets - To emphasize patient-centered design, robust clinical analysis, and the careful selection of physiological, behavioral, and environmental monitoring devices

## Data Availability

Data sharing is not applicable to this article as no new data were created or analyzed in this study.

## Appendix A

This appendix provides a brief description of some popular medical terms and technologies, along with a brief explanation of how AI tools can assist in the health informatics field:

- Digital human twins (DHTs) refer to the digital replicas of biological systems. Digital twins function as data-driven simulations of patient health and treatment responses with scales ranging from cellular mechanisms to whole-body systems, according to Meier-Schellersheim [204]. DHTs can simulate organs, tissues, and entire physiological processes to enable personalized healthcare interventions [98]. The deployment of DHTs relies on data infrastructure that includes cloud computing, communication protocols, medical databases, virtual reality, and AI technologies. There are already successful examples of digital twin use in hospital operations, according to Cheng et al. [205]. AI is essential for managing and interpreting the vast datasets that power these models, and it plays a critical role in refining them to more accurately mirror real humans.
- Disease identification may be handled by classification methods like binary classification, while multi-class classification methods enable more nuanced differential diagnoses, according to De Silva et al. [30]. Machine learning models are able to perform many classification tasks satisfactorily. For example, machine learning has been used to analyze routine clinical and lab data to predict the illness of endoscopic disease [206].
- Electronic health records (EHRs) are digital systems for documenting healthcare services and have become fundamental tools in medical care and system management [207]. Their global uptake has accelerated sharply. In the U.S., EHR adoption surged from 10% in 2008 to nearly 96% by 2017. Similarly, China reported an adoption rate exceeding 85%, according to Liang et al. [208].
- Internet of Medical Things (IoMT) encompasses interconnected systems that collect, transmit, and analyze medical data using sensors, communication modules, and data processors. As societies face the challenges of an aging population, the shift toward home-based care is emerging as a practical and sustainable approach with IoMT [36]. The Internet of Things (IoT) emerges as the most widely adopted communication framework [73]. IoT technologies allow medical staff to monitor and treat patients remotely. Connected IoT devices can collect real-time health data, allowing prompt medical responses, according to Ngankam et al. [209]. Through IoT, AI-enhanced wearables can provide clinicians with a more holistic picture of a patient’s health status with real-time patient assessment [96,210]. When integrated with IoT, IoMT can enhance service delivery [211].
- Pharmacometrics enhances care by modeling drug effects and disease progression. Key challenges include inconsistent data quality in pharmacometrics and the absence of unified standards, according to Cucurull-Sanchez et al. [212]. AI can play a pivotal role in analyzing complex datasets to forecast disease progression, identify viable pharmacological targets, and tailor treatment to individual profiles.
- Precision medicine focuses on customizing treatments to each patient’s genetic profile, lifestyle, and environment. Precision medicine relies heavily on complying genomic data with EHRs to tailor medical treatment to individual patients. The success of precision medicine hinges on advancements in big data analytics and AI technologies, according to Abul-Husn et al. [213]. It can tailor diagnostic and therapeutic approaches. Central to this concept is the principle of the so-called five rights: delivering the right treatment to the right patient at the right time, in the right dose, and through the right route [94]. AI emerges as a tool for crafting more effective antibiotic regimens, tracking resistance genes, identifying emerging mutations, and boosting the precision of monitoring systems. Notable AI advancements in precision medicine for neurological disorders have been seen in conditions such as Alzheimer’s, Parkinson’s, Huntington’s, and various neuropsychiatric illnesses, according to Gupta and Kumar [115].
- Radiomics enables the extraction of quantitative data from medical images, capturing minute details that often go unnoticed by the human eye [95]. Machine learning can process these high-dimensional imaging features to noninvasively predict histological subtypes, genetic mutations, and patient outcomes. This allows for more refined risk assessment and personalized treatment.
- Virtual health assistants represent a breakthrough in healthcare support tools. Designed to replicate human interactions, they deliver customized care through patient inputs, according to Curtis et al. [214]. These systems often combine chatbots, audio interfaces, and other AI-driven features to assist with symptom evaluation, health guidance, engagement management, and health tracking. Timely alerts and context-aware messaging are areas of growing interest, according to Al Kuwaiti [84]. Machine learning models can guide personalized care interventions.
- Wireless body area networks (WBANs) consist of sensors attached to the human body for monitoring vital health indicators. These systems enable seamless communication between the human body and digital devices via wireless technology. For instance, wearable sensors can track metrics like blood pressure or glucose levels and transmit real-time data to healthcare providers. When combined with wearables, AI models show a strong potential in handling physiological data from wearables to conduct individualized stroke risk analysis and to deliver personalized recommendations, according to Olawade et al. [164]. The adoption of intelligent wearable devices has brought remote chronic disease management for diabetes, hypertension, sleep apnea, and asthma into mainstream practice [215]. These intelligent WBANs can even operate autonomously, capable of making independent judgments, according to Rajasekaran et al. [53].

## Appendix B

This appendix provides a brief description of some popular AI technologies that are frequently used in health informatics:

- Machine learning (ML) is a subset of AI that empowers computing devices to learn and make decisions autonomously, without being explicitly instructed. In other words, machine learning enables systems to learn autonomously from data, refining their performance over time without direct supervision, according to Ghassemi et al. [216]. Machine learning helps refine and analyze data to extract clinically useful insights from high-dimensional inputs [96]. ML models are also adept at detecting patterns and clustering data into meaningful categories [217]. ML plays a vital role in collecting, interpreting, and analyzing medical data, ultimately assisting clinicians in making more informed diagnoses, according to Kaplan et al. [218]. It has increasingly been applied in telehealth and intelligent care systems to aid disease management and support patient self-care [22]. The ML models show promising results in improving predictive accuracy [66]. A range of machine learning models, including support vector machines (SVM), k-nearest neighbors (KNN), and neural networks (NN), have been employed to detect Parkinson’s disease [219]. Machine learning clustering methods have been employed to stratify populations based on their potential health risks [220].
- Deep learning (DL) is a rapidly advancing branch of ML, which is leveraged to interpret complex medical data across diverse healthcare fields, according to Varghese et al. [221]. Its applications span from natural language processing in healthcare to diagnostics, telemedicine, precision medicine, and medical imaging [222]. Esteva et al. [223] found that deep learning models designed for skin cancer detection performed on par with expert dermatologists. DL models have shown high accuracy in detecting coronary artery disease, although they require large datasets and struggle with smaller ones [224]. Zain Eldin et al. [225] introduced a novel deep learning framework that may push the boundaries of neuroscience by offering a more refined understanding of brain function, potentially elevating patient care services.
- Generally speaking, modern deep learning models consist of multi-layered neural networks. Neural networks have long been successfully incorporated into surgical diagnostics, like aiding the evaluation of abdominal pain, diagnosing appendicitis, detecting bile duct stones, and assessing glaucoma [226]. These tools can improve diagnostic accuracy, which is essential in healthcare.
- Among the neural network models, convolutional neural networks (CNNs) are very popular and are based on the human visual processing system. CNNs are designed to extract complex features from image data through structured layers that perform specific tasks [227]. These layers apply convolutional filters to detect patterns in input data [228]. They are the backbone of many image-based medical diagnostic systems.
- Lack of interpretability among the ML and DL models limits their real-world application, even though these models have superior performance in healthcare prediction tasks, especially with complex datasets [26]. Key barriers remain, including the opaque “black box” nature of many AI algorithms and concerns about the long-term viability of mHealth and telehealth solutions [17].
- Transformer architecture is a foundational deep learning innovation, which has gained prominence across fields like visual computing, natural language processing, and speech analytics [229]. Its application in healthcare is growing rapidly, with use cases in analyzing electronic health records, interpreting clinical scans, and tracking COVID-19, according to Shamshad et al. [230].
- Large language models (LLMs) are advanced AI models known for their massive scale and capabilities that go beyond what smaller models can achieve [231]. Built using deep learning frameworks and trained on enormous datasets, these models are proficient in natural language processing tasks. LLMs are capable of proficiently drafting clinical documentation, ensuring consistency and quality while also saving time [21]. LLMs demonstrate promise in a range of healthcare tasks, including supporting clinical processes, summarizing text, streamlining documentation, and enabling multilingual interactions [61]. Because of their advanced language understanding, LLMs are now able to perform many human-like language tasks [232]. However, these models are also prone to producing misleading or fabricated content, with this phenomenon known as hallucination.
- Generative AI (GenAI) models represent a powerful subset of machine learning able to produce synthetic data that closely resembles actual datasets. Capable of producing new content, like text, images, and molecular designs, based on learned data patterns [233], GenAI holds the potential to transform several areas in healthcare, like creating substitute datasets for rare disease research, supporting AI-driven drug discovery, and powering intelligent virtual health assistants [79,234]. When integrated into clinical settings, GenAI may offer personalized support for physicians. Its ability is valuable in medical settings, where high-quality training data may be scarce, making these models essential for data augmentation and simulation. Technologies like generative adversarial networks and large language models enable the creation of highly realistic outputs, according to Alqahtani et al. [235]. For example, models like GPT-4 can generate fluent natural language, code, and other structured data [236]. The capabilities of generative AI are used in healthcare to synthesize patient datasets, support research, and accelerate drug development [234]. A major hurdle for GenAI is that stringent privacy regulations severely limit the data sharing needed for effective training, according to Rudrapatna and Butte [237]. Complicating matters further, healthcare datasets are often inconsistent, created for different purposes, and lack standardization, according to Hersh et al. [238].
- Generative Pre-trained Transformer (GPT) is an advanced language model that processes and generates human-level sentences by leveraging large-scale pre-training, followed by task-specific fine-tuning. In the medical field, its versatility spans applications including educational content development, decision support, patient communication, documentation, and invoicing processes [239].
- Natural language processing (NLP) is an AI subfield centered on interpreting and generating human language. Leveraging deep learning, NLP technologies are able to sift through massive volumes of data to identify meaningful patterns, which is especially relevant in healthcare, where much of the information is embedded in free-text formats. NLP technologies can interpret patient responses to facilitate faster, more accurate diagnoses. Jerfy et al. [33] conducted a systematic review of 27 studies highlighting the implementation of natural language processing in healthcare. The review emphasized that analyzing large, unstructured datasets presents substantial opportunities for NLP.
- Chatbots are powered by AI and natural language processing to simulate human-like conversation and hold promise for improving healthcare delivery [240]. Chew [241] noted the growing integration of AI chatbots in clinics. Their use can streamline data collection, ease the burden on healthcare staff, and enable more focused interactions between patients and clinicians, according to Hindelang et al. [24]. Chatbots can also assist in clinical trials by addressing patients’ questions and analyzing patient-generated data to detect risk factors [147].
- ChatGPT has proven helpful in generating differential diagnoses, simulating clinical cases, and assisting with examination preparation, thus enhancing learning efficiency during the early stages of medical training, according to Safranek et al. [93]. ChatGPT’s GPT-4 has shown promise in producing context-aware surgical documentation for ophthalmology, like operative notes for cataract procedures. Waisberg et al. [242] showed that their approach with GPT-4 can achieve this while safeguarding patient confidentiality, as no private data is required to generate templated notes. Before ChatGPT’s release in late 2022, only a small fraction (7%) of AI applications in nursing and midwifery reported measurable real-world benefits, according to O’Connor et al. [92]. This is in agreement with the common historical trend which many technologies fall short initially and only fulfill their promise after iterative improvements [243]. Nevertheless, with rapid advancements in LLMs and ChatGPT, many of these potentials are now being realized. ChatGPT has shown utility in answering patient queries, assisting with decision-making, supporting clinical trials, and enhancing patient education [97]. Nonetheless, concerns persist regarding the originality of responses, data privacy, factual accuracy, embedded bias, and legal implications.
- Explainable AI (XAI) has drawn increasing attention for its capacity to make complex AI models more transparent [70]. XAI not only helps organizations comply with legal mandates for transparency but also ensures that end users can understand the rationale behind AI decisions through intuitive interfaces [72]. In the past few years, XAI techniques have been increasingly applied to breast cancer research, according to Gurmessa and Jimma [18].

## Appendix C

This appendix presents more details about the reviews listed in Table 1. The ones that focus on clinical decision support are:

- Murtaza et al. [13] conducted a review covering seventy articles that explored various techniques for generating synthetic medical data with built-in privacy safeguards. The review evaluated different synthetic data approaches, highlighting their advantages and limitations in various cases.
- Yang et al. [15] highlighted eight applications for electronic medical records in precision medicine. Nine critical areas facing difficulties due to the rapid development of big data technologies in precision medicine were identified. These include data warehousing and management, electronic health records, biomedical imaging informatics, AI-assisted surgical planning and procedure improvement, omics data analysis, health surveillance systems, knowledge graphs, public health informatics, and issues surrounding data security and privacy.
- Nti et al. [1] examined the intellectual structure underpinning the field of technology for sustainable healthcare (TSH). Their review covered a total of 18,729 publications. The review highlighted a steady upward trend in research related to TSH. Frequently used technologies include health informatics, medical informatics, healthcare, and artificial intelligence.
- Van Der Vegt et al. [16] reviewed 37 studies focused on the use of artificial intelligence to predict real-time clinical deterioration of adult patients. Their analysis explored the effectiveness of these AI tools and identified various factors affecting the embedding of AI into clinical systems. The findings suggest that predictive systems have the potential to enhance clinical practices and contribute to reduced patient mortality rates.
- Yang et al. [19] examined 37 studies centered on over-sampling strategies designed to manage multi-class imbalances in medical and other datasets, aiming to further increase the accuracy of AI-powered predictive models. Their review revealed a growing preference for hybrid resampling techniques, which merge different methods to address data imbalance issues.
- Monson et al. [28] conducted a large-scale review of 3939 articles to examine the use of AI in ophthalmology and vision science. A co-occurrence network analysis showed that diabetic retinopathy was frequently linked with keywords such as “deep learning,” “machine learning,” and “artificial intelligence,” indicating a strong research emphasis on using AI for diagnostic and triage purposes in eye care. The importance of promoting open-source code sharing to increase global collaboration and enable greater participation from underrepresented regions in AI development was highlighted.
- Krefting et al. [29] conducted a total of six studies examining current trends in OSA, along with two focusing specifically on the application of clinical decision support systems (CDSS) in OSA management. Emerging evidence suggests that incorporating artificial intelligence with CDSS has the potential to enhance patient-centered care through earlier and more accurate diagnosis.
- De Silva et al. [30] explored how speech signal analysis can support the clinical decision process, reviewing 72 studies involving primary data on neurological conditions. These investigations employed traditional machine learning, deep learning, and hybrid models to derive clinical insights from speech features. Future research should prioritize creating interpretable and reliable representations of speech-based digital biomarkers to facilitate replication and accelerate progress in this field.
- Ognjanović et al. [31] examined developments in biomedical and health informatics (BMHI), analyzing 17 articles. Their synthesis identified five main areas of focus: biomedical and healthcare informatics, data handling in health contexts, the role of nurses in informatics, educational and accreditation standards, and ethical, legal, and security challenges. Recurring themes included the use of AI for individualized medical decision processes and the importance of system compatibility. The critical need to approach BMHI research with a people-focused lens to ensure relevance and effectiveness was highlighted.
- Jerfy et al. [33] conducted a systematic review of 27 studies highlighting the implementation of natural language processing in healthcare. The review emphasized that analyzing large, unstructured datasets presents substantial opportunities for NLP to uncover insights. However, this advancement comes with limitations, such as data privacy concerns, significant computational requirements, and challenges in aligning NLP tools with current AI systems.
- Sheng et al. [41] reviewed five studies focused on using AI for detecting oral cancer. They noted that analyzing biomarkers in various bodily fluids could be pivotal in screening healthy individuals. Sheng et al. projected that AI will increasingly support smart, preventative oral health systems, improving early detection accuracy and treatment response. Suggested directions for future exploration include biomarker identification, pathogen detection, monitoring occlusal force, drug delivery mechanisms, tooth whitening techniques, and enhanced human–machine interfaces.
- Marengo and Santamato [44] reviewed 63 studies focusing on the role of quantum computing in healthcare. Their findings revealed two main areas of interest: the use of quantum computing to enhance AI applications in medicine and its role in strengthening the security of healthcare data. Quantum algorithms were found to offer significant advantages, such as optimizing complicated diagnostic processes and boosting efficiency for health-related data analysis. However, challenges like the need for scalable algorithms and the integration of fault-tolerant quantum hardware remain critical obstacles to clinical implementation.
- Almalki and Karunamoorthi [45] examined 17 studies relating to the Zika virus outbreak. Their analysis suggested that artificial intelligence can contribute meaningfully to various phases of outbreak management, including diagnosis, monitoring, and emergency response. Almalki and Karunamoorthi advocated for the thoughtful incorporation of AI technologies to strengthen public health infrastructure and enhance outbreak preparedness.

Among the above reviews on clinical decision support, advanced predictive diagnosis analysis with AI tools is very popular, appearing in studies of Yang et al. [15], Van Der Vegt et al. [16], Yang et al. [19], Monson et al. [28], Krefting et al. [29], Sheng et al. [41], and Marengo and Santamato [44]. Privacy issues and data security are the main concerns in the studies of Murtaza et al. [13], Yang et al. [19], and Jerfy et al. [33].

The reviews in Table 1 that focus on patient care are summarized as follows:

- Asan et al. [17] investigated how artificial intelligence can play a transformative role in healthcare by making it more patient-friendly. Asan et al. examined the influence of AI on consumer health informatics (CHI), emphasizing its importance for advancing patient-centered care. Their review covered 20 studies exploring AI-driven CHI systems, patient health outcomes, and user perceptions. These systems were divided into three categories: mobile health (65%), robotics (25%), and telemedicine (10%).
- Peek et al. [23] highlighted that the shift toward digital health services, driven by AI and data sharing, has transformed the healthcare landscape after the onset of the COVID-19 pandemic. A total of 27 articles on AI and data sharing were covered. It is encouraged for practitioners to collaborate across different disciplines, with the goal of identifying scenarios where ML can offer genuine medical contributions.
- Hindelang et al. [24] conducted a structured review of 18 studies exploring the role of chatbots in taking medical histories. Their findings suggest that chatbots can support clinical decision-making by efficiently collecting patient data through structured questioning, enhancing patient engagement and satisfaction. Future research is needed to improve chatbot algorithms, expand their emotional responsiveness, and adapt them for broader clinical use to maximize their potential in modern healthcare.
- Ambalavanan et al. [27] explored how AI technologies can help understand long COVID. Fourteen studies were reviewed that applied hierarchical classification techniques to categorize symptoms associated with long COVID. The combination of long COVID as a variable within digital health informatics platforms and the use of standardized frameworks to guide its management was advocated. The findings underscore the ongoing need for research and technological solutions aimed at supporting patients experiencing long-term effects of COVID-19.
- Awan et al. [32] reviewed five key studies that explored the intersection of social media, crowdsourced data, and AI in public health. Awan et al. examined the role of social media in enhancing public health initiatives. They argued that platforms like Twitter and Facebook can be instrumental in promoting health education, elevating research standards, and boosting the overall quality of healthcare services. A future direction is about integrating AI, including Generative Pre-Trained Transformers, with social media analytics to address ongoing challenges in health informatics.
- Chan et al. [37] analyzed 33 studies on noninvasive blood glucose monitoring (NIBGM), concluding that artificial intelligence can effectively forecast glucose trends using noninvasive inputs while offering improved comfort and user-friendliness for patients. However, Chan et al. emphasized the importance of addressing regulatory, safety, and quality concerns in future developments of AI-powered monitoring technologies.
- Bragazzi and Garbarino [4], in their review of eight studies, explored the emerging field of sleep infodemiology and infoveillance. They advocated for future research focused on the systematic tracking and analysis of sleep-related information and misinformation, utilizing advanced tools like machine learning, NLP, and data science.
- Denecke et al. [38] identified both enablers and barriers to adopting AI in participatory health informatics (PHI) in a review of 38 studies. The main advantages included enhanced diagnostic precision, more efficient patient management, and greater individual engagement in health decisions. On the other hand, legal uncertainties and data privacy issues were noted as key obstacles to wider AI integration.
- Sharma and Sharma [39] conducted a review of 12,974 articles. In 2013, just 153 publications were identified that linked artificial intelligence to healthcare. However, from 2019 to 2023, the number of such publications surged dramatically, signaling rapid advancement and heightened interest in the field. Commonly associated keywords include machine learning, big data, cloud computing, the Internet of Things, deep learning, and data mining. Notably, the term “privacy” emerged frequently, though it does not refer to a technological concept itself. The study emphasized AI’s transformative potential to enhance patient outcomes, streamline healthcare operations, and spark innovation across the sector. Future research must emphasize the development of AI systems that are ethically grounded, transparent, and interpretable.
- Quinones et al. [40] explored how machine learning can predict patient-reported outcome measures (PROMs) following spinal surgery. Their review, covering 25 studies, highlighted PROMs as critical indicators of how spine-related conditions affect both physical and mental well-being. The findings showed that ML models can accurately forecast these outcomes. However, the lack of a unified evaluation standard across studies remains a significant limitation. Future work should focus on creating standardized outcome metrics and consistent benchmark frameworks to support the comparability and effectiveness of ML models.
- An et al. [43] explored how artificial intelligence is being applied in the context of obstructive sleep apnea (OSA), which is a common condition marked by repeated airway blockages during sleep, leading to reduced oxygen levels and excessive daytime fatigue. Their comprehensive review covered 867 studies. Frequently mentioned keywords included machine learning, ECG (electrocardiography), and deep learning. Major research themes encompassed diagnosis, patient identification, personalized treatments, prognosis prediction, telehealth applications, and disease management. An et al. suggested that future progress will rely on advanced AI systems capable of integrating data from diverse sources to deliver more accurate, patient-specific, and accessible care. Interdisciplinary collaboration was also highlighted as essential to enhance AI model reliability and maintain medical applicability.
- Albari Burayk et al. [46] investigated seven case studies on diabetic ketoacidosis and hyperosmolar hyperglycemic state, in addition to current clinical protocols and treatment procedures. Their review emphasized that health informatics improves patient outcomes by streamlining data management, supporting clinical processes, and enabling remote care. They also noted that AI shows a strong potential to refine diagnostic precision, facilitate continuous monitoring, improve patient engagement, and enhance cooperation among health service providers.

Among the above reviews on patient care, AI tools like AI-powered chatbots and GenAI were helpful for increasing patient satisfaction/convenience in studies of Asan et al. [17], Hindelang et al. [24], Chan et al. [37], Sharma and Sharma [39], and Albari Burayk et al. [46]. Several studies by Peek et al. [23], Awan et al. [32], An et al. [43], and Albari Burayk et al. [46] emphasized the need of collaborating across different disciplines. Privacy issues are a main concern in the studies of Chan et al. [37], Denecke et al. [38], and Sharma and Sharma [39]. The need for consistent benchmark frameworks to support the comparability of ML models was emphasized in the study by Quinones et al. [40].

The reviews in Table 1 that focus on electronic health records are summarized as follows:

- Chang et al. [20] explored the advantages of artificial intelligence, the Internet of Things, and personal health records in improving healthcare delivery. They reviewed 24 systematic reviews and meta-analyses. Their findings indicate that AI contributes positively to clinical results, often outperforming traditional diagnostic methods with accuracy scores ranging from 0.83 to 0.99.
- Wu et al. [21] conducted a systematic review and meta-analysis involving 37 studies about burnout among healthcare professionals due to electronic health record usage. Their analysis found a significant link between EHR use and increased burnout. The study suggests that artificial intelligence may serve as a remedy by streamlining EHR operations and reducing the clerical burden on clinicians through task automation.
- Caterson et al. [26] reviewed 76 studies focusing on explainable AI (XAI) applications in electronic health record analysis. The most frequently used method was Shapley Additive Explanations (SHAP), found in 63 studies, followed by partial dependence plots (PDPs) in 11, and Locally Interpretable Model-Agnostic Explanations (LIME) in 8. A major gap identified in this literature is the insufficient reporting of methods and a lack of rigorous assessment of model validity and reliability. It was suggested that future research should focus on evaluating how XAI tools are deployed within clinical environments.
- Monteith et al. [47] reviewed five studies investigating the intersection of artificial intelligence, cybersecurity threats, and healthcare. Their findings raised concerns over the increasing use of AI by malicious actors. Medical records are especially vulnerable due to their high black-market value. Monteith et al. warned that AI is being leveraged to design more sophisticated cyberattacks that can evade detection software, generate synthetic deepfake content for fraud, and automate widespread, coordinated attacks. These developments have significantly broadened the threat landscape in healthcare cybersecurity.
- Shimu et al. [50] examined nine studies exploring the role of health informatics in addressing drug addiction, with a particular focus on the integration of electronic health records and artificial intelligence. The research highlighted the potential of digital platforms, such as EHRs, telehealth systems, and clinical decision support tools, to closely monitor individual patient data and deliver personalized care. AI was shown to be effective in analyzing large datasets to identify patterns in addictive behavior, thereby supporting tailored interventions and treatment planning.

Among the above reviews on patient care, AI tools are very helpful on electronic health records, according to Chang et al. [20], Wu et al. [21], and Shimu et al. [50]. The need for consistent benchmark frameworks to support the comparability of ML models was emphasized in the study by Caterson et al. [26], while the concerns about privacy/security issues are discussed in the study by Monteith et al. [47].

The reviews in Table 1 that focus on hospital management are summarized as follows:

- Scandiffio et al. [25] investigated how mentoring and coaching strategies support healthcare professionals in adopting digital tools, including artificial intelligence. Their review included 19 studies examining such programs aimed at promoting the integration of digital technologies. It was emphasized that future research should examine the traits and qualifications of effective mentors or coaches that lead to successful skills development. Additional important considerations include efficient resource distribution, thoughtful program design, the quality of the mentor–mentee communication, data privacy issues, and the importance of setting clear objectives for attendants.
- Dhabliya et al. [34] explored the advantages of AI in the healthcare domain, pointing to enhanced diagnostic accuracy, better treatment outcomes, and optimized resource use. They reviewed ten articles on AI-driven medical diagnostics and stressed the importance of developing policies that foster innovation while safeguarding patient rights. Future directions call for refining AI algorithms and implementing ethical evaluation frameworks tailored to public health.
- Glicksberg and Klang [35] narrowed their focus to the most impactful AI research in health and life sciences over the past three years. They reviewed 50 highly cited papers, identifying major trends and categorizing key themes. These included clinical applications such as diagnosis, virtual care, medical training, disease classification, and personalized treatment planning. The authors stressed that as AI technologies evolve rapidly, there must be a parallel effort to ensure ethical oversight and robust regulation.
- Ariza-Colpas et al. [48] reviewed 1134 studies examining the application of artificial intelligence in understanding long-term symptoms associated with COVID-19. Their analysis found that AI provides novel and powerful tools for detecting intricate patterns and risk indicators that may elude conventional analytical methods. In addition to aiding diagnosis and treatment, AI can enhance the allocation of healthcare resources by prioritizing care for high-risk patients. Ariza-Colpas et al. emphasized the importance of cross-disciplinary cooperation to bring together clinicians, epidemiologists, data scientists, and health policymakers for unlocking the full potential of AI in improving clinical outcomes and shaping public health strategies.
- Damar et al. [49] conducted a large-scale review of 32,046 publications, including 50 focused specifically on public health informatics, to explore the influence of artificial intelligence, machine learning, robotic process automation, and virtual reality on healthcare systems. Their findings advocate for the inclusion of AI-related subjects such as machine learning and deep learning in undergraduate health informatics curricula, reflecting the inherently interdisciplinary nature of the field and its growing importance in modern healthcare.

The reviews in Table 1 that focus on remote patient monitoring are summarized as follows:

- Christopoulou [22] reviewed 18 articles to investigate how ML is utilized in evidence-based telehealth and smart care. The review found that embedding ML with evidence-based healthcare practices can improve individualized care, enhance early detection of health issues, facilitate better communication between patients and providers, increase performance, and reduce expenses. However, the need for additional systematic reviews, meta-analyses, and evaluations to unlock the full benefits of ML in healthcare delivery was emphasized.
- Seth et al. [36] conducted an extensive review of 158 publications, revealing that prevalent topics within the Internet of Medical Things (IoMT) include cloud-based solutions, RESTful APIs, Wi-Fi, data gateways, JSON formatting, MQTT protocols, and WebSocket technology. The study suggested that future investigations should prioritize collaborative frameworks and technological integrations aimed at enhancing system interoperability.

Among the above reviews on remote patient monitoring, Christopoulou [22] emphasized the need for meta-analyses to unlock the full benefits of ML in healthcare delivery, while Seth et al. [36] talked about the importance of common frameworks for better integration and collaboration.

The reviews in Table 1 that focus on genomic medicine are summarized as follows:

- Demir and Yegin [42] investigated AI’s role in disease prediction and genomic data analysis, surveying 32 studies spanning genetics, medicine, and data science. Their review underscored AI’s capacity to pinpoint risk factors, interpret genetic mutations, and guide personalized treatment planning. Personalized healthcare seeks to improve both outcomes and patient satisfaction by customizing medical approaches to the individual’s specific needs, preferences, and genetic traits. Demir and Yegin concluded that AI holds enormous potential to revolutionize both healthcare delivery and genetic research, but also called attention to the need for strong ethical and legal safeguards in its implementation.

The reviews in Table 1 that focus on diagnostic imaging are summarized as follows:

- Hua et al. [14] reviewed 31 studies to identify the primary factors that influence how healthcare practitioners in medical imaging perceive AI. The study emphasizes that for AI tools to gain broader acceptance, they must be designed with a strong people-focused approach. This means focusing not just on technical accuracy but also on making systems usable for individuals with varying levels of AI knowledge, aligning with real-world medical operations, and adapting to institutional and professional values around ethics, security, and culture. It was highlighted that the acceptance of AI in healthcare differed markedly from its acceptance in the consumer or business field, where the stakes for human well-being are generally lower.
- Gurmessa and Jimma [18] reviewed the XAI applications in diagnosing breast cancer through mammography and ultrasound imaging. Their analysis included 14 studies that utilized XAI methods for this purpose. Most of the reviewed studies highlighted challenges related to the datasets used. While findings suggest that XAI can improve diagnostic accuracy and help minimize human mistakes, current evidence remains insufficient to determine the most effective XAI approach. Future research is necessary to draw definitive conclusions.

Among the above reviews on genomic medicine and diagnostic imaging, Demir and Yegin [42] and Hua et al. [14] talked about concerns related to privacy/security issues.

## Appendix D

This appendix presents more details about the reviews listed in Table 2. The ones that focus on clinical decision support are:

- Allemailem et al. [55] examined the role of multi-sensor technologies in regular surveillance of patient health, analyzing 15 relevant studies. Their analysis highlighted that AI can significantly augment these sensor systems, particularly in forecasting health trends and supporting predictive analytics. One of the main challenges lies in processing the complex data produced by magnetic sensors, which requires advanced signal processing techniques to derive actionable medical insights. Future research may prioritize proactive healthcare solutions that both enhance the results of healthcare interventions and streamline medical services.

The reviews in Table 2 that focus on patient care are summarized as follows:

- Nag et al. [56] explored how AI technologies are reshaping patient care through the lens of emotional intelligence and person-centered interventions. Their review, which encompassed 123 publications, found that AI has played a pivotal role in customizing treatment approaches, facilitating prompt detection of mental health conditions, and supporting evidence-based therapeutic decisions. Key concerns persist around ethical use, data privacy, and uncovering hidden biases in AI algorithms. There will be considerable potential in integrating varied data sources, like diagnostic imaging, electronic health records, wearables, and patient self-reports, to develop more holistic, personalized care frameworks.

The reviews in Table 2 that focus on electronic health records are summarized as follows:

- Erandathi et al. [54] reviewed 102 research articles focused on using artificial intelligence to predict complications associated with diabetes mellitus. Their findings suggest that AI-driven prognostic models, especially those incorporating diverse risk factors, are becoming increasingly popular due to their efficiency and affordability. It is recommended to develop a standardized set of features that can serve as a benchmark for evaluating diabetes-related risks and circumstances.

The reviews in Table 2 that focus on hospital management are summarized as follows:

- Khalifa and Albadawy [5] conducted a comprehensive review exploring how artificial intelligence is reshaping diagnostic imaging in healthcare. Their analysis spanned 30 scholarly articles and highlighted AI’s growing impact in areas such as image processing, clinical interpretation, operational workflows, individualized care, and assistance for medical decisions. Khalifa and Albadawy emphasize the importance of future initiatives focused on developing ethical standards, equipping healthcare workers with appropriate training, and ensuring that AI advancements remain focused on patient needs.
- Adibi et al. [52] reviewed how AI contributes to healthcare in smart environments where the spaces were equipped with sensors, digital tools, and location-aware services. Adibi et al. analyzed eight articles and concluded that AI helps these environments adapt intelligently to individual needs, thereby improving comfort, personalization, and operational efficiency. However, the protection of data privacy and security remains a major concern. Interdisciplinary collaboration and adherence to ethical principles to ensure that smart environments are developed responsibly and sustainably are advocated.

The reviews in Table 2 that focus on remote patient monitoring are summarized as follows:

- Rajasekaran et al. [53] reviewed developments in wireless body area networks (WBANs), covering topics such as communication protocols, security measures, authentication techniques, routing standards, and the embedding with AI. By incorporating AI, WBAN systems now offer more accurate, context-aware health assessments tailored to individual users. The study identified eleven distinct applications of WBAN technology.

Among the above reviews on health analytics, advanced predictive diagnosis analysis with AI tools is very popular [52,53,54,55,56]. Privacy issues and data security are a main concern in the studies of Adibi et al. [52] and Rajasekaran et al. [53], while Erandathi et al. [54] and Khalifa and Albadawy [5] talked about the importance of common benchmarks and ethical standards, respectively.
